# Kikuchi-Fujimoto disease presenting in a patient with SARS-CoV-2: a case report

**DOI:** 10.1186/s12879-021-06048-0

**Published:** 2021-08-03

**Authors:** Samuel D. Racette, Borislav A. Alexiev, Michael P. Angarone, Ajay Bhasin, Kaitlin Lima, Lawrence J. Jennings, Senthil Balasubramanian, Akihiro J. Matsuoka

**Affiliations:** 1 Department of Otolaryngology-Head and Neck Surgery, Feinberg School of Medicine, Northwestern University, 676 N. St. Clair Street, Suite 1325, Chicago, IL 60611 USA; 2 Department of Pathology, Feinberg School of Medicine, Northwestern University, Chicago, IL USA; 3 Department of Medicine, Division of Infectious Diseases, Feinberg School of Medicine, Northwestern University, Chicago, IL USA; 4 Department of Medicine, Division of Hospital Medicine, Feinberg School of Medicine, Northwestern University, Chicago, IL USA; 5 Department of Pediatrics, Division of Hospital Based Medicine, Feinberg School of Medicine, Northwestern University, Chicago, IL USA; 6 Department of Medicine, Division of Rheumatology, Feinberg School of Medicine, Northwestern University, Chicago, IL USA; 7 NorthShore University Health System, Evanston, IL 60201 USA; 8 Roxelyn and Richard Pepper Department of Communication Science Disorders, School of Communication, Northwestern University, Evanston, IL 60208 USA; 9 Hugh-Knowles Hearing Center, Evanston, IL 60208 USA

**Keywords:** COVID-19, SARS-CoV-2, Kikuchi-Fujimoto Syndrome, Cervical lymphadenopathy

## Abstract

**Background:**

We present a yet to be described association of SARS-CoV-2 infection with Kikuchi-Fujimoto disease.

**Case presentation:**

A 32-year-old physician with history of SARS-CoV-2 infection presented to the emergency department with 2 weeks of fever, chills, and right sided cervical lymphadenopathy. He was treated empirically for presumed folliculitis with worsening of symptoms leading to repeat presentation to the emergency department.

Extensive workup was unrevealing of an infectious cause and needle biopsy of the lesion was unrevealing. An excisional lymph node biopsy revealed follicular hyperplasia with necrotic foci showing abundance of histiocytes at the edge of necrosis with CD8 predominance of T-cells. Final diagnosis was deemed to be Kikuchi-Fujimoto disease. Antibiotic therapy was discontinued, and the patient’s symptoms resolved with steroid therapy and expectant management.

**Conclusions:**

This is the first report of a patient developing Kikuchi-Fujimoto disease following SARS-CoV-2 infection. Clinicians should be aware of Kikuchi-Fujimoto disease as a possibility when approaching patients with hyper-inflammatory states who present with cervical lymphadenopathy.

## Background

The World Health Organization declared COVID-19 a global pandemic in January of 2020 [1]. Since that time many associations with this newly described viral illness have become clinically apparent. The medical literature grows daily and insight into the sequelae of the SARS-CoV-2 virus similarly is expanding at unprecedented pace. As of now there is yet to be a case of Kikuchi-Fujimoto described in the literature associated with SARS-CoV-2 infection. Kikuchi-Fujimoto remains a rare inflammatory disease previously associated with multiple conditions. SARS-CoV-2 is a disease which has become known for leading to devastating sequalae as a result of an induced hyperinflammatory state. Additionally, it is known that health care professionals are at higher risk of contracting SARS-CoV-2 and therefore further understanding of this virus is vitally important [2].

## Case presentation

**Fig. 1:**
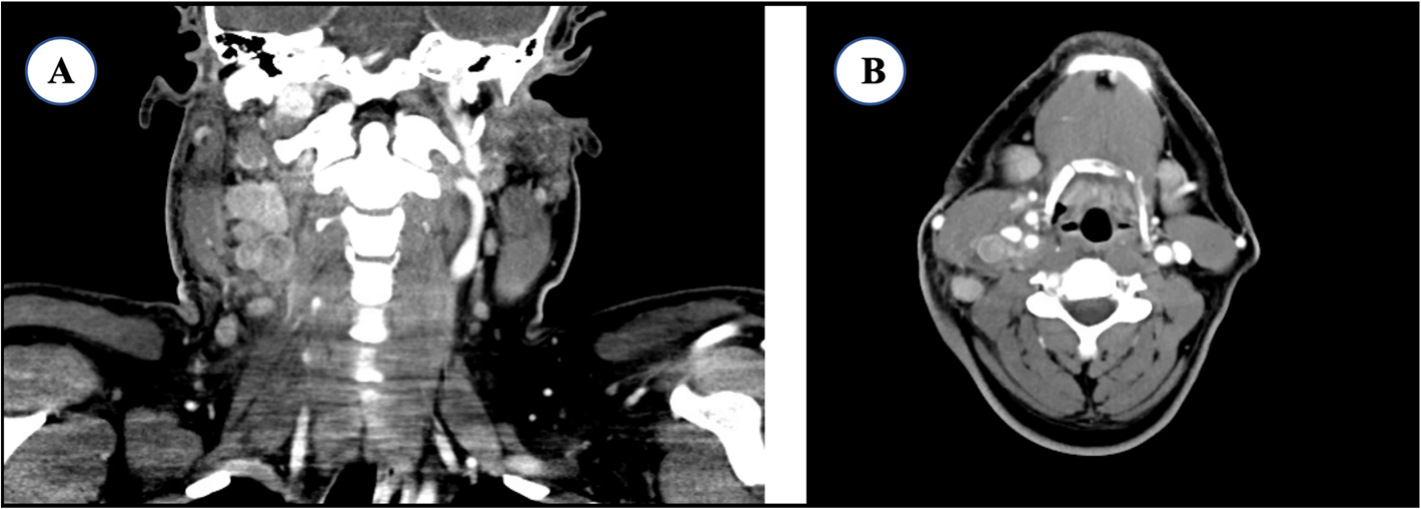
Representative coronal (**a**) and axial (**b**) cuts of a CT neck with contrast displaying right sided cervical lymphadenopathy throughout levels 2-4 along the jugular chain of lymph nodes

A 32-year-old male physician of Indian descent with a history of hypertension, latent tuberculosis treated with 4 months of rifampin completed 1 month prior to presentation, and resolved symptomatic SARS-CoV-2 infection diagnosed 3 months prior and not requiring hospitalization presented to the Emergency Department. His chief complaints at this time were 2 weeks of fever, chills, and neck swelling which began after a haircut. He had completed a course of doxycycline followed by trimethoprim-sulfamethoxazole for presumed folliculitis without improvement. On presentation to the hospital the patient complained of right neck swelling, worsened fatigue, myalgias and fevers. A CT with contrast of the neck was obtained and the patient was discharged with a plan for outpatient biopsy (Fig. 1). However, he returned to the Emergency Department 3 days later due to persistent symptoms and ongoing fever. At that time was noted to be febrile to 101.6 degrees Farenheit, tachycardic to 124 bpm, leukopenic with WBC 2.8 K/UL, absolute neutrophil count of 0.8 K/UL, and LDH elevated at 289 unit/L. Rapid SARS-CoV-2 nucleic acid amplification test was negative. He was admitted for further workup and treatment.

On admission a core needle biopsy of the right cervical lymphadenopathy was performed which revealed necrotic tissue and was inadequate for diagnosis. He was started on cefepime for febrile neutropenia and cyclic fevers. Extensive workup showed no evidence of HIV, Syphilis, Parvovirus, Mononucleosis, CMV, Adenovirus, Toxoplasmosis, Bartonella, and no growth on bacterial, fungal, or acid-fast bacilli blood cultures. Autoimmune panel including anti-ds-DNA antibody and anti-Smith antibody were negative. Due to inadequate sample on needle biopsy, concern for worsening B symptoms, and need to rule out lymphoma; an excision lymph node biopsy was planned. A SARS-CoV-2 PCR test was repeated at this time per operating room protocol and was found to be positive.

**Fig. 2:**
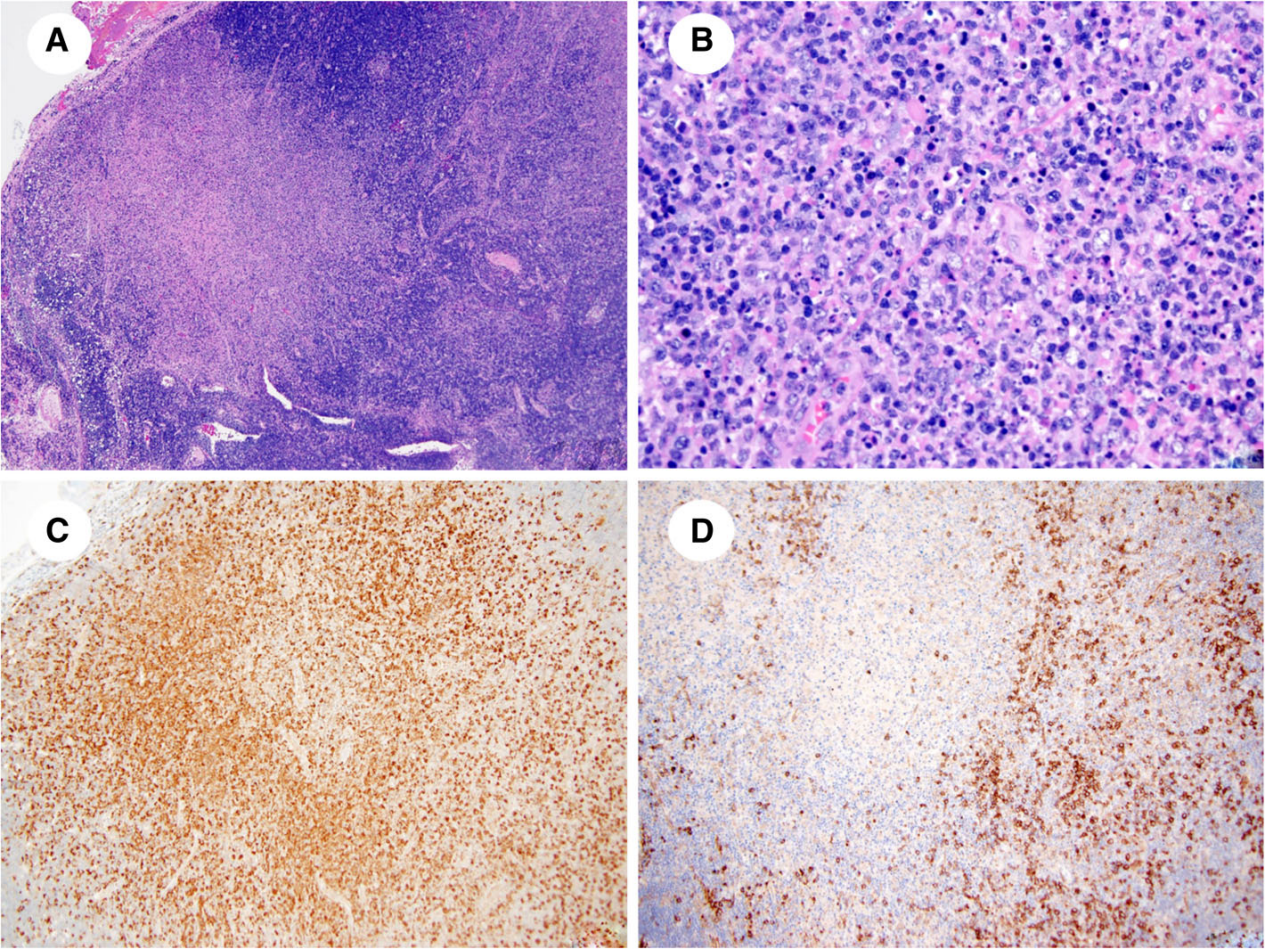
1**A**. Necrotizing lymphadenitis with KFD-like features. Note expanded paracortex and wellcircumscribed area of necrosis. Hematoxylin-eosin stain, x40. 1**B**. Necrotizing lymphadenitis with KFD-like features. Note abundant karyorrhectic nuclear debris and a large accumulation of histiocytes. Neutrophils are absent. Hematoxylin-eosin stain, x200. 1**C**. Necrotizing lymphadenitis with KFD-like features. Histiocytes are positive CD68. CD68 stain, x100. 1**D**. Necrotizing lymphadenitis with KFD-like features. Clusters of plasmacytoid dendritic cells are highlighted by CD123 stain. CD123 stain, x100

Following SARS-CoV-2 testing an excisional cervical lymph node biopsy was completed using appropriate PPE for a SARS-CoV-2 positive patient. The lymph node was submitted for histologic analysis which showed partially preserved architecture with follicular hyperplasia. The paracortex was expanded with focal, well-circumscribed areas of necrosis (Fig. 2a). Necrotic foci show abundant karyorrhectic nuclear debris and a large accumulation of histiocytes at the edge of necrosis (Fig. 2b). Among the histiocytes, there were scattered small lymphocytes, activated T cells, and some plasma cells. Neutrophils and eosinophils were absent. At the edge of necrotic areas, there were clusters of plasmacytoid dendritic cells, as well as immunoblasts. No granulomas were identified.

The histiocytes were positive for myeloperoxidase, CD68 (Fig. 2c), and CD4. The lymphocytes were mostly CD3-positive T cells demonstrating a predominance of CD8 compared with CD4, with very few CD20-positive B cells. Plasmacytoid dendritic cells were highlighted with CD123 (Fig. 2d). CMV and SARS-CoV-2 stains for the lymph node were negative and further microbiologic assessment of the lymph node including culture of the tissue for aerobic and anaerobic bacteria, acid fast bacilli, and fungus was negative.

This result was deemed to be consistent with Kikuchi-Fujimoto Disease. The antibiotics were discontinued, and given the persistent fever and hospitalization the patient was discharged on oral prednisone (0.5 mg/kg) with plan for inflammation control. The patient had a favorable response to steroid treatment and since discontinuation has remained asymptomatic with resolution of all symptoms.

## Discussion and conclusions

SARS-CoV-2 infection has been declared a Public Health Emergency of International Concern by the WHO on January 30th, 2020. Since this time the risks of transmission to healthcare workers has been well documented. Our patient was a physician known to be infected with SARS-CoV-2 in March with resolution of symptoms in April. The repeat positive test in July may indicate a false positive, viral shedding, DNA presence without infectivity or a new SARS-CoV-2 infection. There are case reports of patients with persistent SARS-CoV-2 positive tests despite clinical recovery. These individuals are thought to be chronic shedders of virus despite the resolution of symptoms [1]. The significance to the patient’s health of the persistently positive SARS-CoV-2 test is currently unknown. Clinically, infection of SARS-CoV-2 has been characterized clinically by a myriad of well described symptoms along with generalized lymphopenia and T-cell dysregulation. In particular, there appears to be greater CD-8 T-cell activation compared to CD-4 cells in patients who developed mild disease such as our patient [2]. Increased CD-8 T-cell activity is also a hallmark of KFD.

Kikuchi-Fujimoto disease is a rare clinical entity known as histiocytic necrotizing lymphadenitis characterized most often by several weeks of tender lymphadenopathy (most commonly cervical) and fever in individuals of Asian descent under the age of 40. The patient described above meets this characterization as he is an Indian male below the age of forty who suffered with those exact symptoms. The etiology of KFD has been postulated to be either infectious or autoimmune in nature, but no study has identified a specific pathogen associated with the disease syndrome. Previous case reports have identified the disease in patients with other autoimmune conditions [3]. The case described above is the first such case of a patient known to have a previous SARS-CoV-2 infection who later developed KFD.

There is no specific test which is diagnostic for KFD. Pathology from lymph node specimens remain essential for diagnosis with excisional biopsy preferred as core biopsy samples are frequently non-diagnostic due to low sample yield. The differential diagnosis includes infectious lymphadenitis of different etiologies, autoimmune lymphadenopathy (primarily SLE lymphadenopathy), and non-Hodgkin lymphoma. SLE lymphadenopathy is the most difficult to resolve as it is histologically and immunohistochemically indistinguishable from KFD. Importantly, lymphoma must be ruled out as both entities share similar clinical presentations and at times histologic features [4].

Several infectious etiologies, including viruses, tuberculosis, histoplasmosis, leprosy, cat-scratch disease, and *Yersinia enterocolitica* can also present with paracortical expansion with necrosis and histiocytic infiltrate, thus mimicking KFD morphologically. Infectious mononucleosis usually shows marked follicular hyperplasia and paracortical expansion with an increase in immunoblasts and scattered Hodgkin- and Reed-Sternberg–like cells. Single-cell apoptosis and foci of necrosis are also commonly seen. Epstein-Barr virus–encoded RNA in situ hybridization is usually strongly and diffusely positive and resolves the diagnostic dilemma [4]. Special stains and immunohistochemical stains are sometimes helpful in identifying the infectious agents. The majority of these stains were not utilized in this case given previous negative serologic testing.

Active tuberculosis infection can present in a similar manner as this patient presented and given his history of latent tuberculosis it was important to rule this out as a causative factor. He was known to have completed a 4 month course of rifampin only 1 month earlier and was asymptomatic throughout treatment. The acid-fast bacilli blood cultures on admission to the hospital were negative. Lymph node excisional biopsy revealed no granulomas and no evidence of acid-fast bacilli. Additionally, there was no growth of acid-fast bacilli on culture of the patient’s tissue. Although there was a history of tuberculosis infection; there was no histologic or microbiologic evidence of extrapulmonary tuberculosis infection despite robust testing. Active tuberculosis infection as etiology of this patient’s symptoms was deemed to be less likely.

The lymph node specimen was also tested for SARS-CoV-2 and was found to be undetectable. Experience in our laboratory shows that in known SARS-CoV-2 positive individuals there is often a failure to amplify the virus, via PCR, in tissues other than the lung or liver due to low viral load. The lack of detection within the lymph node specimen does not rule out that this patient had active SARS-CoV-2 infection, but makes the diagnosis of Kikuchi-Fujimoto disease possible.

This is the first case report on a patient presented developed Kikuchi-Fujimoto disease following SARS-CoV-2 infection. Clinicians should be aware of Kikuchi-Fujimoto disease as a possibility when approaching patients after SARS-CoV-2 infection who present with cervical lymphadenopathy.

## Data Availability

Datasharing is not applicable as no datesets were generated during this study as the materials represent healthcare information obtained from one individual patient’s chart.

## Abbreviations

SARS-CoV-2: Severe acute respiratory syndrome-Coronavirus-2
KFD: Kikuchi Fujimoto Disease
PCR: Polymerase Chain Reaction
PPE: Personal Protective Equipment
RT-PCR: Real Time-Polymerase Chain Reaction
CMV: Cytomegalovirus
HIV: Human Immunodeficiency Virus
